# MoDPepInt: an interactive web server for prediction of modular domain–peptide interactions

**DOI:** 10.1093/bioinformatics/btu350

**Published:** 2014-05-28

**Authors:** Kousik Kundu, Martin Mann, Fabrizio Costa, Rolf Backofen

**Affiliations:** ^1^Bioinformatics Group, Department of Computer Science, 79110 Freiburg and ^2^Centre for Biological Signalling Studies (BIOSS), 79104 Freiburg, University of Freiburg, Germany

## Abstract

**Summary**: MoDPepInt (Modular Domain Peptide Interaction) is a new easy-to-use web server for the prediction of binding partners for modular protein domains. Currently, we offer models for SH2, SH3 and PDZ domains via the tools SH2PepInt, SH3PepInt and PDZPepInt, respectively. More specifically, our server offers predictions for 51 SH2 human domains and 69 SH3 human domains via single domain models, and predictions for 226 PDZ domains across several species, via 43 multidomain models. All models are based on support vector machines with different kernel functions ranging from polynomial, to Gaussian, to advanced graph kernels. In this way, we model non-linear interactions between amino acid residues. Results were validated on manually curated datasets achieving competitive performance against various state-of-the-art approaches.

**Availability and implementation:** The MoDPepInt server is available under the URL http://modpepint.informatik.uni-freiburg.de/

**Contact**: backofen@informatik.uni-freiburg.de

**Supplementary information**: Supplementary data are available at *Bioinformatics* online.

## 1 INTRODUCTION

Protein–protein interactions are often mediated by modular protein domains in eukaryotes and play an essential role in diverse biological processes such as signal transduction, cellular growth and cell polarity (Pawson and Nash, 2003). Modular domains that specifically bind with short linear peptides are known as peptide recognition modules. Each domain family recognizes peptides with specific characteristics. For example, phosphotyrosine (pY)-containing peptides, proline-rich peptides and C-terminus peptides are recognized by SH2, SH3 and PDZ domains, respectively. However, individual domains from the same family show different binding specificity. Accurate models that can help understand the mechanisms responsible for the highly selective binding affinity are therefore of interest. Recently, several high-throughput techniques, such as protein microarray, phage display and SPOT synthesis, have been developed, which can detect the binding specificity of various modular domains. However, efficient bioinformatics tools are needed to extract meaningful knowledge from the enormous amount of data produced.

To this end, we used state-of-the-art machine learning approaches to build support vector machine models that can accurately predict binding specificity. We have collected into a unified web-based system called MoDPepInt (Modular Domain Peptide Interaction), three different tools: SH2PepInt, SH3PepInt and PDZPepInt for three different modular domains, namely, SH2, SH3 and PDZ (Kundu *et al.*, 2013a,b; Kundu and Backofen, 2014). Currently, we offer single domain models for 51 SH2 human and 69 SH3 human domains, and multidomain models for 226 PDZ domains across human, mouse, fly and worm. To assess the quality of our models, we have used manually curated interaction data achieving competitive performance against various state-of-the-art approaches.

In summary, MoDPepInt unique features include (i) a domain-peptide prediction system for SH2, SH3 and PDZ in a single platform and (ii) the largest number of modeled domains (see Supplementary Table S1).

## 2 APPLICATION AND FUNCTIONALITY

### 2.1 Input

All tools have a unified input format. Query sequences (up to a maximum number of 500) can be supplied either in a FASTA format or using UniProt database accession numbers. PDZPepInt offers predictions also for domains that are newly developed and/or not comprised in the original 226 PDZ domains: the unknown query domain should be supplied in FASTA format. Multiple query domain sequences can also be provided.

### 2.2 Filters

Several filters are available to increase predictive accuracy. SH2 domains generally recognize phosphotyrosine (pY) residues of binding proteins. For this reason, in SH2PepInt, we offer a *phosphotyrosine* filter that only considers those peptides whose tyrosine phosphorylation has already been experimentally verified and reported in PhosphoSitePlus database (Hornbeck *et al.*, 2012).

As SH3 domains mainly bind with proline-rich peptides, in SH3PepInt, we offer a *proline-rich* filter that uses 31 regular expressions to select proline-rich peptides (Carducci *et al.*, 2012).

PDZ domains have the tendency to bind the unstructured C-terminal regions of binding proteins; hence, in PDZPepInt, we offer a filter to select for *intrinsically unstructured/disordered regions* based on the IUPred algorithm (Dosztanyi *et al.*, 2005), which selects five C-terminal residues with IUPred scores >0.4 (Akiva *et al.*, 2012).

Finally, a *cellular localization* filter is available for all tools. This filter considers only those interactions where both the protein containing the peptide and the protein containing the modular domain have the same cellular localization according to the Gene Ontology Database (Ashburner *et al.*, 2000).

### 2.3 Processing and output

An internal queuing system (which currently uses 40 computation nodes) balances the submitted jobs in parallel. MoDPepInt is implemented in C++, perl and shell scripting, with runtimes typically ranging in the order of few minutes.

**Fig. 1.**
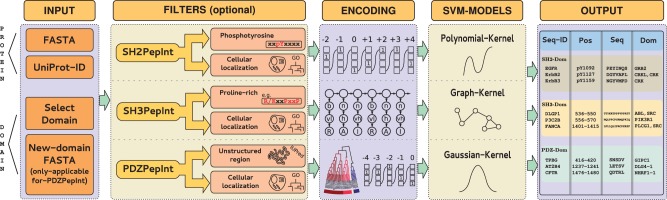
Schematic representation of the MoDPepInt pipeline

The output for all three tools is formatted as a downloadable table. We report for each domain–ligand protein interaction pair (i) the sequence ID, (ii) the ligand binding position, (iii) the ligand binding sequence and (iv) the ligand binding domains. See Figure 1 for the schematic representation of the MoDPepInt pipeline.

## 3 DISCUSSION

MoDPepInt collects three protein–protein interaction predictive models that can be efficiently tuned using data derived from various high-throughput experimental techniques and thus do not require structural information as in Brannetti *et al.*, 2000 and Hou *et al.*, 2008, 2012. The resulting models exhibit significant performance improvement in comparison with other existing tools. The main sources of performance improvement are due to the following: (i) non-linear modeling and advantage over linear PWM models (Obenauer *et al.*, 2003), (ii) balanced discriminative training and (iii) datasets pooling.

SH2PepInt uses polynomial kernels, and it is trained on additional high-confidence negatives obtained via semisupervised techniques.

SH3PepInt uses graph kernels on a complex representation of both the peptide sequence and the aligned domains. The adoption of a graph-type representation allows the inclusion of the physico-chemical properties of amino acids, which increases the generalization capacity of the models. Furthermore, the method does not need any prior alignment of the peptides. This is a big advantage because poly-proline-rich peptides are hard to align.

PDZPepInt uses Gaussian kernels, and it is trained on interaction data from additional highly related domains. Using pooling from closely related domains allows to leverage the limited information available for some domains and to extrapolate to unseen, but alignable, novel domains.

Once trained, all models can be used to efficiently scan entire proteomes to identify novel interactions with typical runtimes of few minutes.

In addition, we offer a meta-web server to be used in non-expert mode that submits the input simultaneously to all tools and displays a summary of the main results. For performance comparisons, details on the novelty of the methods and description of the meta-web server, see Supplementary Information.

*Funding*: This work was funded by the Bundesministerium für Bildung und Forschung (e-bio; FKZ 0316174A to R.B.) and the Excellent Initiative of the German Federal and State Governments (EXC 294 to R.B.).

*Conflict of interest*: none declared.

## Supplementary Material

Supplementary Data
