# Supplementary material for: MoDPepInt: an interactive web server for prediction of modular domain–peptide interactions
Source: Bioinformatics. 2014 May 28;30(18):2668–9. doi: 10.1093/bioinformatics/btu350 (PMC4155253; doi:10.1093/bioinformatics/btu350)
Supplement: Supplementary Data [file supp_30_18_2668__index.html]

MoDPepInt: An interactive webserver for prediction of modular domain-peptide interactions — MoDPepInt: an interactive web server for prediction of modular domain–peptide interactions — MoDPepInt: an interactive web server for prediction of modular domain–peptide interactions — Supplementary Data 

# MoDPepInt: an interactive web server for prediction of modular domain–peptide interactions

## Supplementary Data

files

**Files in this Data Supplement:**

- Supplementary Data - pdf file
