# Supplementary material for: MoDPepInt: an interactive web server for prediction of modular domain–peptide interactions
Source: Bioinformatics. 2014 May 28;30(18):2668–9. doi: 10.1093/bioinformatics/btu350 (PMC4155253; doi:10.1093/bioinformatics/btu350)
Supplement: Supplementary Data [file supp_btu350_supplementary.pdf]

MoDPepInt: An interactive webserver for prediction of modular domain-peptide interactions
# - **Supplementary Material** -

Kousik Kundu [1], Martin Mann [1], Fabrizio Costa [1] and Rolf Backofen [1,2]

[1]Bioinformatics Group, Department of Computer Science, University of Freiburg, Germany,
[2]Centre for Biological Signalling Studies (BIOSS), University of Freiburg, Germany

1 Domain coverage of the available tools:

MoDPepInt currently offers the largest number of modeled domains and a high coverage SH2, SH3 and PDZ domain-peptide prediction system in a single platform. Here, we discuss the domain coverage of other available tools in Table S1.

Table S1: Domain coverage of the available tools. The table clearly shows that the MoDPepInt has higher domain coverage than other tools.

**Tools**	**Domains**			**Total**	**Pubmed Ref.**
	**SH2**	**SH3**	**PDZ**		
ScanSite	14	13	-	27	12824383
SMALI	76	-	-	76	18424801
DomPep	97	-	189	286	22003397
SH3Hunter	-	16	-	16	16870929
**MoDPepInt**	51	69	226	**346**	23690949, 23813002, 24564547

2 Performance comparison:

In our studies, we employed a support vector machines (SVMs) using different kernel functions (e.g. Gaussian, polynomial and sophisticated graph kernels) to build predictive single-domain models for 51 SH2 domains and 69 SH3 domains and multi-domain models for 226 PDZ domains across the species that include human, mouse, fly and worm. We compared our results with several state- of-the-approaches. In the following sections we describe the performances of our three different tools (i.e. SH2PepInt, SH3PepInt and PDZPepInt). Results are derived from the following publications:

- Kousik Kundu, Fabrizio Costa, Michael Huber, Michael Reth, and Rolf Backofen Semi-Supervised Prediction of SH2-Peptide Interactions from Imbalanced High-Throughput Data PLoS One, 8(5), pp. e62732, 2013.
- Kousik Kundu, Fabrizio Costa, and Rolf Backofen A graph kernel approach for alignment-free domain-peptide interaction prediction with an application to human SH3 domains Bioinformatics, 29(13), pp. i335-i343, 2013.
- Kousik Kundu and Rolf Backofen Cluster based prediction of PDZ-peptide interactions BMC Genomics, 15 Suppl 1 pp. S5, 2014.

2.1 SH2PepInt performance:

All the models were built on support vector machine (SVM) with polynomial kernel. A stratified 5 fold cross-validation technique has been used to evaluate the predictive performance of each SH2 domain. We compute the area under the ROC curve (AUC ROC) and the area under the precision and recall curve (AUC PR) (see Figure S1). The following results are derived from the [8].

We compare our results with two state-of-the-art tools: SMALI [9], and an energy model approach [15]. SMALI could be applied to 45 test sets as it does not have model for the other 6 SH2 domains. Our model achieves an average AUC ROC of 0.83 and average AUC PR of 0.93 (see Figure S1), outperforming the other two approaches: SMALI achieves AUC ROC of 0.71 and AUC PR of 0.87; the energy model achieves AUC ROC of 0.62 and AUC PR of 0.81. We note that SMALI achieves a very high specificity (0.95 on average) in all 45 SH2 domains when the proposed threshold is used (i.e. relative SMALI score 1), however this comes at the expenses of a very poor sensitivity (0.26 on average).

We also tested our approach with SMALI on a manually curated and reliable database of SH2- peptide interactions called PhosphoELM [3]. We could not test energy model, since there is no specific threshold that can determine the class. On this dataset the performance of SMALI is 112 correct interactions predicted over a total of 335 interactions (26 domains, SMALI does not have models for LCP2 and SOCS2 domains), while our approach identifies 213 true interactions (see Figure S2). In particular, we correctly predicted all the interactions predicted by the SMALI except two interactions for NCK1 and SRC SH2 domain each. Note that we have taken care to exclude all the interaction data in the PhosphoELM database from our training sets (unfortunately this cannot be done for the SMALI tool since we could use only the pre-trained version).

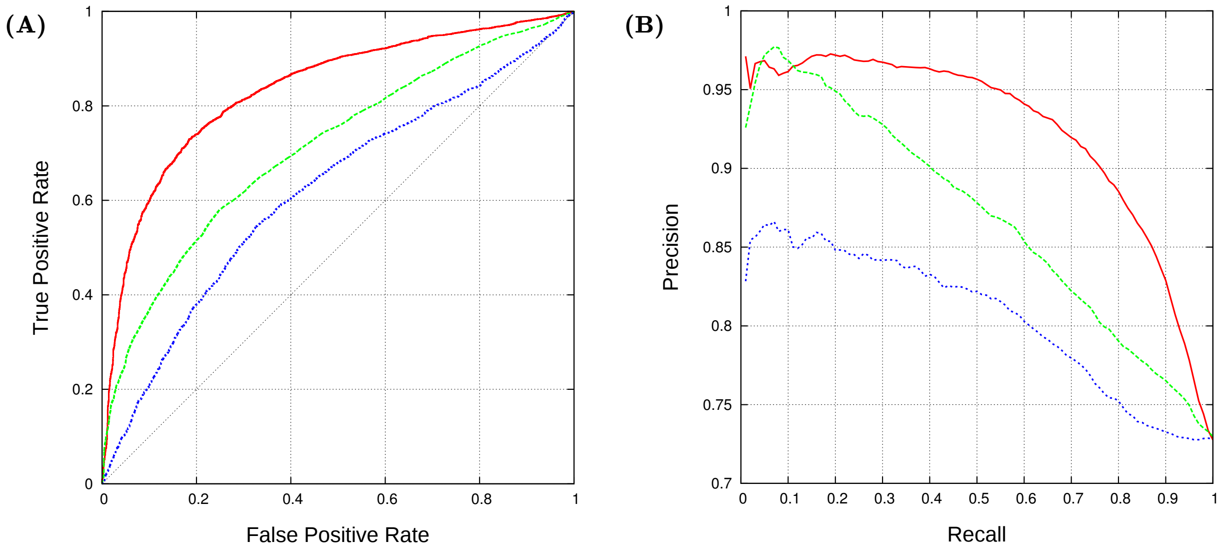

Figure S1: Comparison of AUC ROC and precision-recall curve of three different approaches. (A) Showing the comparison of the AUC ROC for the SVM performance (solid red line), the SMALI performance (dashed green line) and the performance of energy model (dotted blue line). This figure clearly indicates the SVM performance with 0.83 AUC ROC is significantly higher than the SMALI and energy model approaches with 0.71 and 0.62 AUC ROC respectively. (B) Showing the comparison of the precision-recall curve for the SVM performance (solid red line), the SMALI performance (dashed green line) and the performance of energy model (dotted blue line). In this case the SVM performance with 0.93 precision-recall curve is higher than the SMALI and energy model approaches with 0.87 and 0.81 precision-recall curve respectively. The figure is taken from [8].

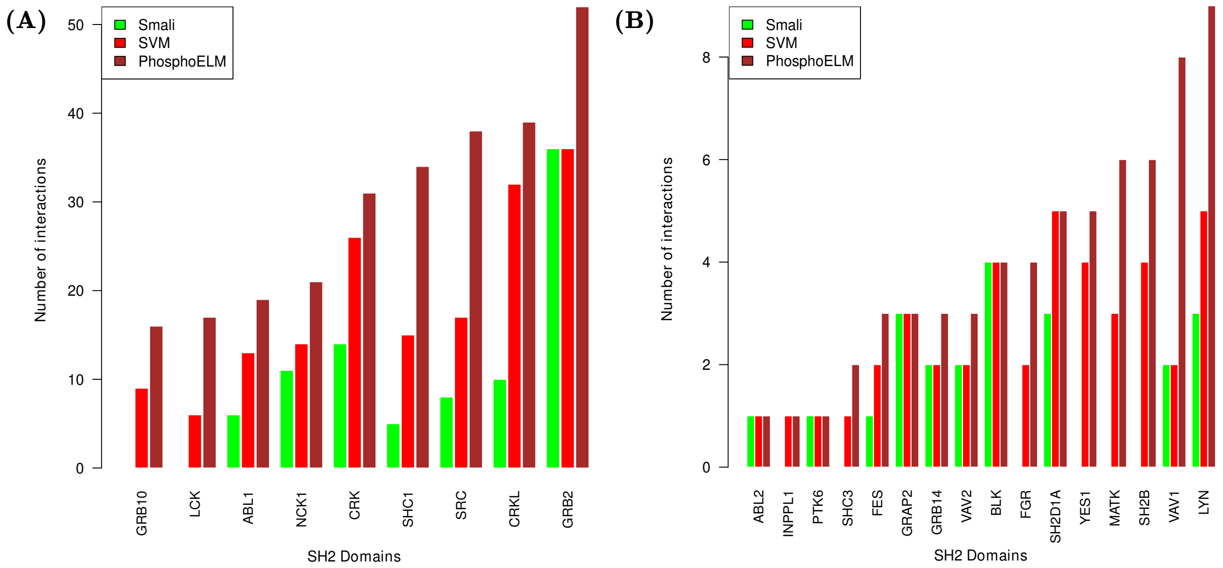

Figure S2: Performance evaluation on manually curated database, PhosphoELM [3]. (A,B) Performance of SMALI and our program on the experimentally validated data. In both (A and B) case the brown bars indicate the actual experimentally validated interactions for individual SH2 domains where the red and green bars indicate the predicted interactions by SVM models and SMALI respectively. (A) Showing those SH2 domains having at least 10 interactions in PhosphoELM 9.0 and (B) Showing the SH2 domains having less than 10 interactions in PhosphoELM 9.0 database. The figure is taken from [8].

2.2 SH3PepInt performance:

All the models are based on support vector machine (SVM) with an efficient graph kernel. A stratified 10 fold cross-validation technique has been used to evaluate the predictive performance of each SH3 domain. We compute the area under the ROC curve (AUC ROC) and the area under the precision and recall curve (AUC PR) (see Figure S3). The following results are derived from the [7].

We compare our results with a recently developed tools, called MUSI [5]. Our models achieve an average AUC ROC of 0.94 and AUC PR of 0.73 while using *filtered negatives* and an average AUC ROC of 0.9 and AUC PR of 0.35 while using *non-filtered negatives*, completely outperform MUSI that achieves an average AUC ROC of 0.69 and AUC PR of 0.27 while using *filtered negatives* and an average AUC ROC of 0.58 and AUC PR of 0.04 while using *non-filtered negatives* (see Figure S3).

To test how important the precise information on true negatives is, we built the one-class model for each SH3 domains. The key idea here is to make use of information based primarily on the positive interactions to characterize the binding peptides; instances that are not well recognized by the model are then assumed to be negative. Once again, we operate in the same setup as for the non-filtered negatives experiment. In Figure S3, we report the comparative results with respect to AUC PR and AUC ROC performance measures for all studied SH3 domains. The one-class approach achieves an average AUC PR 0.063 and 0.61 AUC ROC. Although this result is statistically significant (according to a Wilcoxon Matched-Pairs Signed- Ranks Test, with ρ=0.0003), the magnitude of the result let us conclude that using a generative approach to model protein peptide interactions is noncompetitive with respect to discriminative approaches.

In other experiment we combined the 6 similar SH3 domains and built a single model for all 6 SH3 domains. We evaluated the predictive performance of this multi-domain model using a 10-fold cross-validation over the six domain set using the filtered negatives. we report the AUC PR and the AUC ROC for each SH3 domain and MUSI performance. The experimental result confirms our intuitions: sharing information across related domains increases the predictive performance, mainly owing to an increase in sensitivity.

In addition, we took 478 real interactions reported in the manually curated MINT database [11], discarded them from our training set and could recover 397 (i.e. a recall 0.83) interactions.

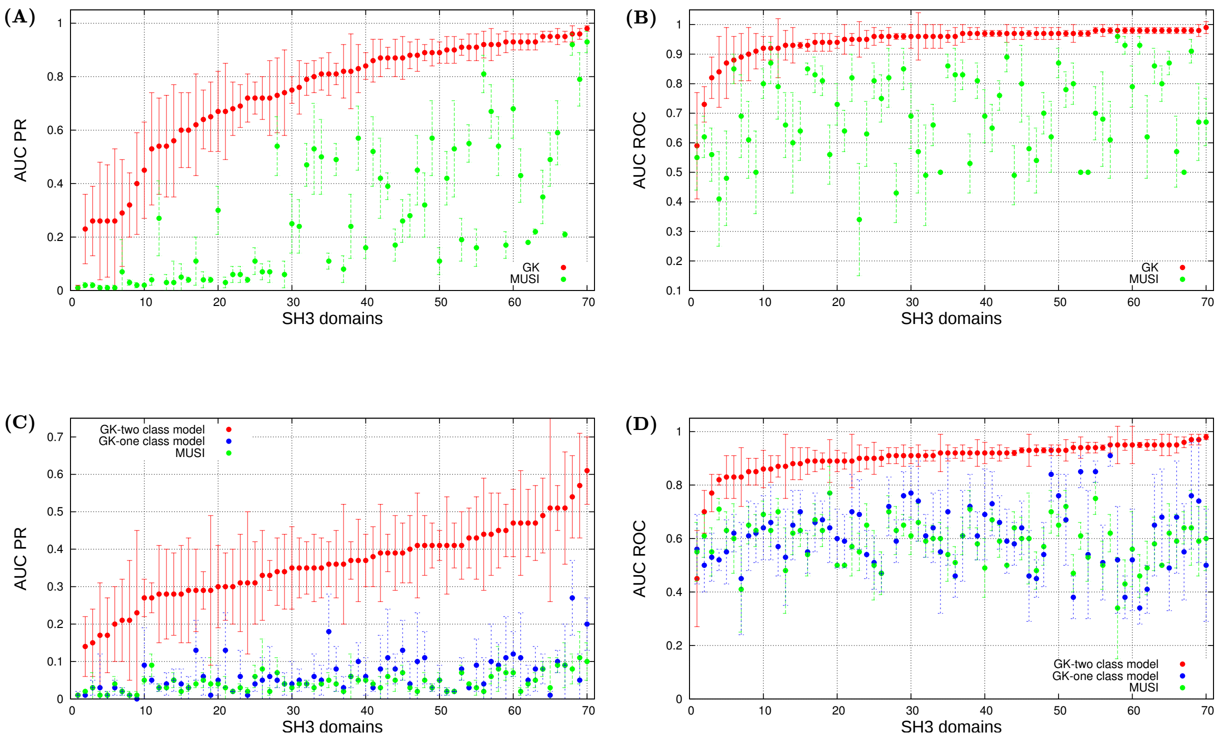

Figure S3: A 10-fold cross-validation performance. (A) and (B) comparison when using filtered negative interactions for Graph Kernel (GK) and MUSI. (C) and (D) comparison with nonfiltered negative interactions for binary class Graph Kernel (GK), one-class Graph Kernel and MUSI. The error bars represent respective standard deviation. The domains are sorted by increasing average performance for the Graph Kernel method. The figure is taken from [7].

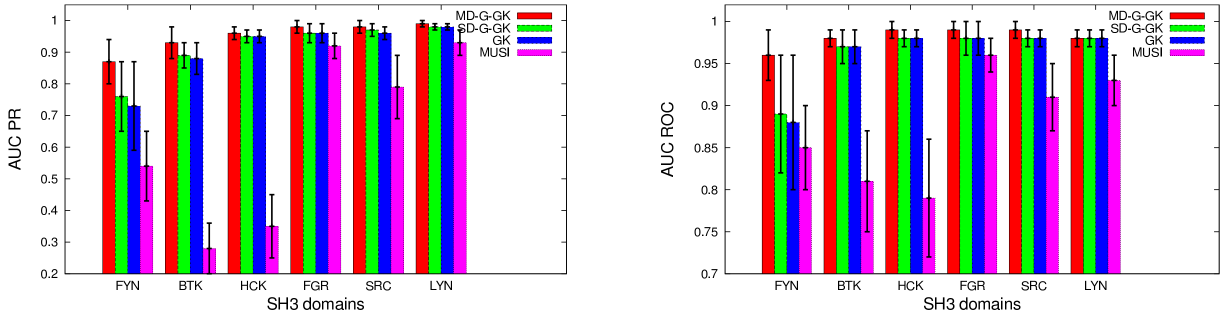

Figure S4: Precision-recall curves and AUC ROC curves for the Multi-Domain Gaussian Graph Kernel (MD-G-GK), the Single Domain Gaussian Graph Kernel (SD-G-GK), the Single Domain Linear Graph kernel (GK) and the MUSI tool for 6 related SH3 domains. The error bars represent respective standard deviation. The figure is taken from [7].

2.3 PDZPepInt performance:

All the models are based on support vector machine (SVM) with a Gaussian kernel. A stratified 5 fold cross-validation technique has been employed to evaluate the predictive performance of PDZ domains. Our models achieve an average AUC ROC of 0.92 and AUC PR of 0.94. The following results are derived from [6].

We compare our results with two state-of-the-art tools, namely MDSM (multi-domain selectivity model) [14] and DomPep [10], on an independent test set. The independent test set contained 493 positive interactions and 3059 negative interactions that involved 74 mouse PDZ domains and 48 peptides [14]. Among them, we used interactions for 50 PDZ domains that were common in all three methods (MDSM, DomPep and our method). We make sure the peptides were not included in our training sets. Our models achieved a true positive rate (TPR) of 0.67, false positive rate (FPR) of 0.14 and AUC ROC of 0.85 with a true-positive/false-positive (TP/FP) ratio of 0.87 outperforming the other two approaches: MDSM achieved TPR of 0.55, FPR of 0.17 and AUC ROC of 0.74 with TP/FP ratio of 0.55; the DomPep achieved TPR of 0.66, FPR of 0.15 and AUC ROC of 0.84 with TP/FP ratio of 0.79 (see Figure S5).

In another experiment, we tested our method with MDSM on a validated dataset. We could not test DomPep since many of the test instances were present in the DomPep training set and hence a fair comparison was not possible. The test data was retrieved from an experimentally validated database, called PDZBase [2]. We compared 20 mouse PDZ-peptide interactions derived from PDZBase that were neither included in MDSM nor in our training set. Out of 20 interactions, we successfully predicted 14 interactions with a true positive rate (TPR) of 0.70, compared to only 4 interactions predicted by MDSM with a true positive rate (TPR) of 0.20. Table S2 lists the scores for all 20 validated interactions as calculated by MDSM and by our method. See [6] for more details.

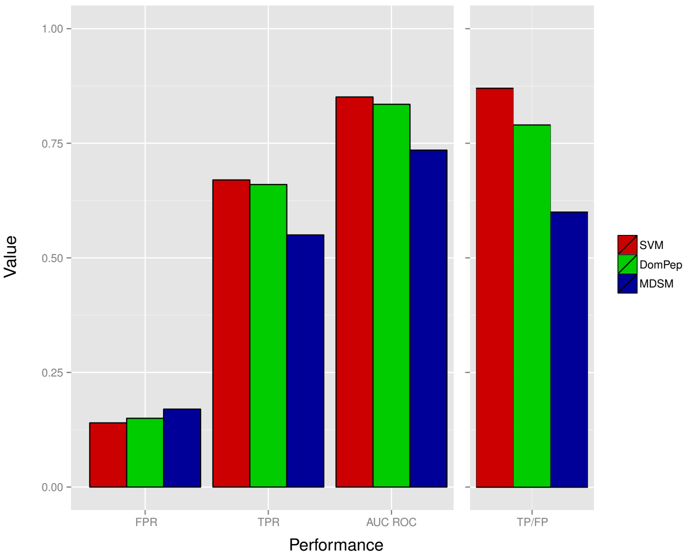

Figure S5: Performance evaluation on an independent test set. Performance comparison of tree different tools. Red, green and blue bars indicate the predicted performances by our tool (SVM), DomPep and MDSM, respectively. The figure clearly shows that our tool (SVM) achieved better performance. This figure is taken from [6]

Table S2: SVM and MDSM scores for experimentally validated interactions derived from PDZBase [2]. A peptide is predicted to bind to a PDZ domain if the score is more than 0 for SVM and more than 1 for MDSM. Bold numbers indicate true positive interactions. This table is taken from [6]

**PDZ domain**	**Peptide**	**SVM score**	**MDSM score**	**Pubmed Ref.**
Cipp-(3/10)	IESDV	**0.44**	-0.7	9647694
Cipp-(3/10)	LESEV	**0.30**	-0.62	9647694
Cipp-(3/10)	QQSNV	**0.29**	-0.78	9647694
Cipp-(3/10)	KEYYV	**0.51**	-0.34	9647694
Dvl1-(1/1)	SETSV	-1.27	-0.74	12490194
Pdlim5-(1/1)	DITSL	-0.24	-0.15	10359609
Erbin-(1/1)	LDVPV	**0.99**	0.61	10878805
Magi-2-(5/6)	KESSL	**1.76**	0.19	10681527
MUPP1-(10/13)	IATLV	**1.00**	0.46	11000240
MUPP1-(10/13)	GKDYV	**1.00**	**1.68**	11689568
NHERF-1-(1/2)	FDTPL	**1.06**	0.01	10980202
LIN-7A-(1/1)	IESDV	**0.33**	0.29	10341223
Lin7c-(1/1)	IESDV	**0.33**	1.00	10341223
ZO-3-(1/3)	GKDYV	**0.99**	0.09	10601346
a1-syntrophin-(1/1)	VLSSV	-1.47	0.16	11571312
PSD95-(1/3)	LQTEV	**0.38**	**1.41**	11937501
PSD95-(1/3)	NETVV	-1.35	**1.19**	12067714
PSD95-(1/3)	GETAV	-1.32	**1.23**	12067714
PSD95-(1/3)	EESSV	-2.23	0.77	11134026
PSD95-(1/3)	RTTPV	**1.00**	0.61	12359873

3 Reasons for performance improvement:

3.1 Non-linear modeling:

Among the shortcomings for current predictive approaches [5, 9, 13] we list:

- limited coverage
- restrictive modeling assumptions as they are mainly based on position specific scoring matrices (PSSM) and do not take into consideration complex amino acids inter-dependencies
- high computational complexity

Previous research has shown that the binding specificity of modular domains [4, 12] is dependent on the correlations between different ligand positions. For this reason we propose domain specific non-linear models where different kernel functions allow higher order correlation between amino acids positions.

3.2 Balanced discriminative training:

Datasets derived from high-throughput experiments usually suffer from a lack of reliable negative interaction data. In our study, we were only able to obtain the negative interaction data from microarray experiments. However the resulting datasets were imbalanced. Other data sources provide only positive interaction data. It is known [1] that machine learning methods work poorly when the dataset is highly imbalanced. In order to ameliorate the problem we have employed a semi-supervised learning approach (SSL). The general strategy of SSL is to learn from a small amount of labeled data and a large amount of unlabeled data. Here, differently from the general problem formulation for SSL, we were interested in using the unsupervised material to have a better characterization only of the minority class (in our case, the negative class). Albeit, there are several approaches for the SSL setting, we have chosen the *self-training* strategy that relies on the good discriminative properties of a base classifier and thus fits

well with our datasets. The initial labeled data is used to train a classifier which then assigns a label to the unlabeled material. The most confident predictions are then iteratively added to the training set and the classifier is retrained. Note that we were only able to apply this semi-supervised technique on SH2 and PDZ peptide interactions as they had at least a few reliable negative interactions [6, 8].

For SH3 domains instead, since there were no reliable negative interactions available, we employed a false negative refinement strategy [7]. The key idea here is to use a generative approach to model each peptide/motif class and select as negative representatives a subset of instances that are not recognized by any specialized model. To better represent the binding specificity of each domain, instead of using a single model, we resort to multiple PWMs, namely, one for each motif class for each SH3 domain. Afterward, we generate a PWM for each motif group and use a sequence homology search algorithm to identify the peptides matching the various PWMs. Finally, for each domain, we selected those peptides that were not recognized by any of the class specific PWMs. See [7] for more details.

3.3 Datasets pooling:

In recent years, an enormous amount of interaction data has been generated by various high-throughput experiments for PDZ interactions thus computational methods have become increasingly more important to analyze these data. One of the major problems here is the different coverage of various domains; for example, in literature only two positive interactions for PDZ 1 and PDZ 2 domains of human DLG2 and DLG4 are known. To overcome this limitation we combined PDZ domains that are similar in substrate specificity. This strategy allowed us to model the binding specificity for related domains as a whole.

4 Meta-webserver:

In addition to the three specialized servers for SH2, SH3 and PDZ, we implemented the meta-webserver MoDPepInt. MoDPepInt is to be used in non-expert mode: a) no parameters need to be set, b) the output comprises predictions for all available domains for SH2, SH3 and PDZ and c) only the 5 most confident predictions for each domain are reported. However, the user can easily select one of the dedicated tools for the same input to access the full prediction results and have a finer control over its parametric setting.

References

[1] P. Baldi, S. Brunak, Y. Chauvin, C. A. Andersen, and H. Nielsen. Assessing the accuracy of prediction algorithms for classification: an overview. *Bioinformatics*, 16(5):412–24, 2000.

[2] Thijs Beuming, Lucy Skrabanek, Masha Y. Niv, Piali Mukherjee, and Harel Weinstein. PDZBase: a protein-protein interaction database for PDZ-domains. *Bioinformatics*, 21(6):827–8, 2005.

[3] Francesca Diella, Cathryn M. Gould, Claudia Chica, Allegra Via, and Toby J. Gibson. Phospho.ELM: a database of phosphorylation sites–update 2008. *Nucleic Acids Res*, 36(Database issue):D240–4, 2008.

[4] David Gfeller, Frank Butty, Marta Wierzbicka, Erik Verschueren, Peter Vanhee, Haiming Huang, Andreas Ernst, Nisa Dar, Igor Stagljar, Luis Serrano, Sachdev S. Sidhu, Gary D. Bader, and Philip M. Kim. The multiple-specificity landscape of modular peptide recognition domains. *Mol Syst Biol*, 7:484, 2011.

[5] T. Kim, M. S. Tyndel, H. Huang, S. S. Sidhu, G. D. Bader, D. Gfeller, and P. M. Kim. MUSI: an integrated system for identifying multiple specificity from very large peptide or nucleic acid data sets. *Nucleic Acids Res*, 2011.

[6] Kousik Kundu and Rolf Backofen. Cluster based prediction of PDZ-peptide interactions. *BMC Genomics*, 15 Suppl 1:S5, 2014.

[7] Kousik Kundu, Fabrizio Costa, and Rolf Backofen. A graph kernel approach for alignment-free domain-peptide interaction prediction with an application to human SH3 domains. *Bioinformatics*, 29(13):i335–i343, 2013.

[8] Kousik Kundu, Fabrizio Costa, Michael Huber, Michael Reth, and Rolf Backofen. Semi-Supervised Prediction of SH2-Peptide Interactions from Imbalanced High-Throughput Data. *PLoS One*, 8(5):e62732, 2013.

[9] Lei Li, Chenggang Wu, Haiming Huang, Kaizhong Zhang, Jacob Gan, and Shawn S-C Li. Prediction of phosphotyrosine signaling networks using a scoring matrix-assisted ligand identification approach. *Nucleic Acids Res*, 36(10):3263–73, 2008.

[10] Lei Li, Bing Zhao, Jun Du, Kaizhong Zhang, Charles X. Ling, and Shawn Shun-Cheng Li. DomPep–a general method for predicting modular domain-mediated protein-protein interactions. *PLoS One*, 6(10):e25528, 2011.

[11] Luana Licata, Leonardo Briganti, Daniele Peluso, Livia Perfetto, Marta Iannuccelli, Eugenia Galeota, Francesca Sacco, Anita Palma, Aurelio Pio Nardozza, Elena Santonico, Luisa Castagnoli, and Gianni Cesareni. MINT, the molecular interaction database: 2012 update. *Nucleic Acids Res*, 40(Database issue):D857–61, 2012.

[12] Bernard A. Liu, Karl Jablonowski, Eshana E. Shah, Brett W. Engelmann, Richard B. Jones, and Piers D. Nash. SH2 domains recognize contextual peptide sequence information to determine selectivity. *Mol Cell Proteomics*, 9(11):2391–404, 2010.

[13] John C. Obenauer, Lewis C. Cantley, and Michael B. Yaffe. Scansite 2.0: Proteome-wide prediction of cell signaling interactions using short sequence motifs. *Nucleic Acids Res*, 31(13):3635–41, 2003.

[14] Michael A. Stiffler, Jiunn R. Chen, Viara P. Grantcharova, Ying Lei, Daniel Fuchs, John E. Allen, Lioudmila A. Zaslavskaia, and Gavin MacBeath. PDZ domain binding selectivity is optimized across the mouse proteome. *Science*, 317(5836):364–9, 2007.

[15] Zeba Wunderlich and Leonid A. Mirny. Using genome-wide measurements for computational prediction of SH2-peptide interactions. *Nucleic Acids Res*, 37(14):4629–41, 2009.
